# Supplementary material for: Separating Neural Oscillations from Aperiodic 1/f Activity: Challenges and Recommendations
Source: Neuroinformatics. 2022 Apr 7;20(4):991–1012. doi: 10.1007/s12021-022-09581-8 (PMC9588478; doi:10.1007/s12021-022-09581-8)
Supplement: Supplementary file 1 — Supplementary file1 (DOCX 1728 KB) [file 12021_2022_9581_MOESM1_ESM.docx]

# Supplementary Information


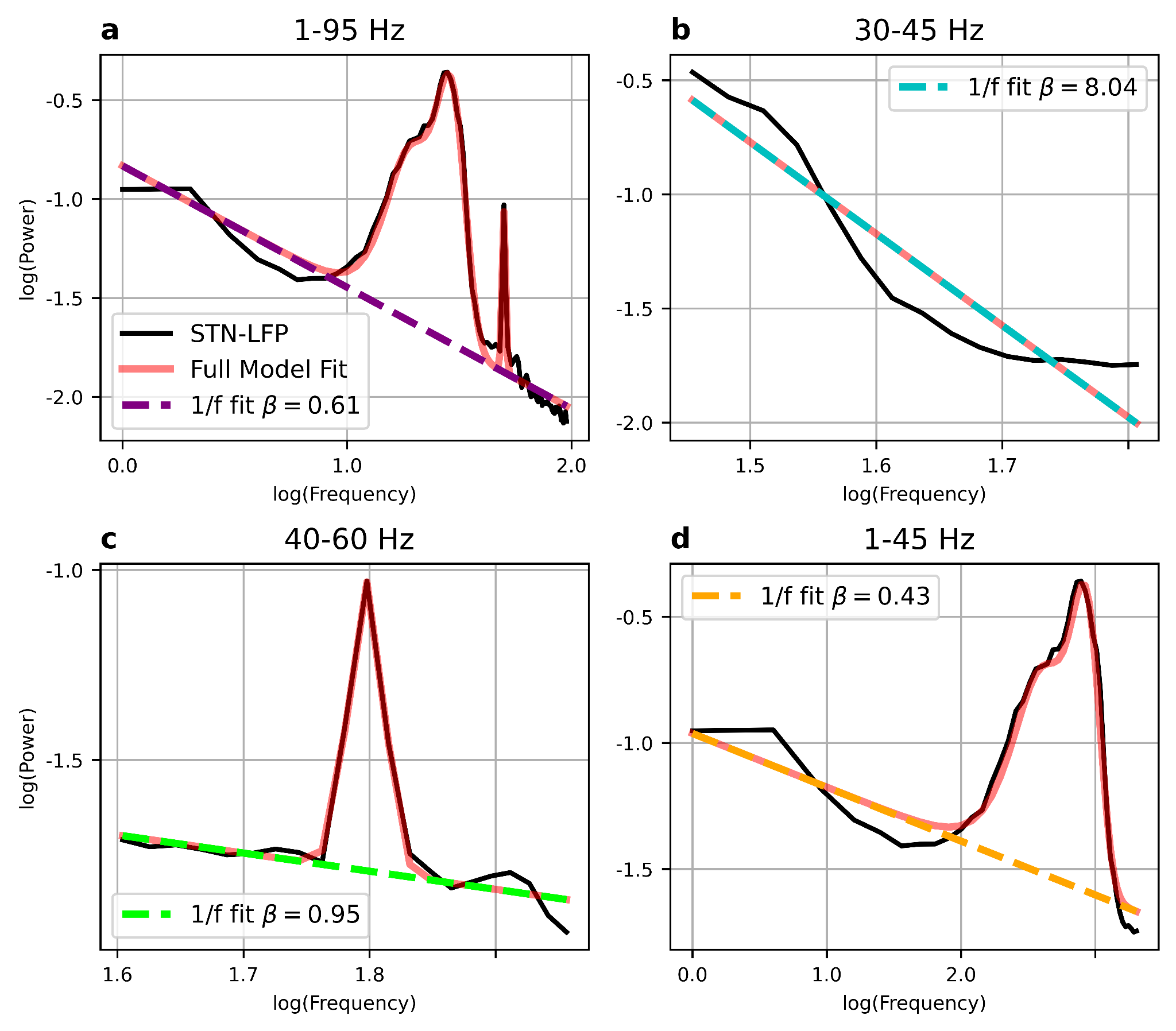


***SI Fig. 1*** *Oscillations crossing fitting range borders. FOOOF fits for the frequency ranges shown in Fig. 2 b). FOOOF parameters: a) max_n_peaks=0, 30–45 Hz, b) max_n_peaks=1, 40–60 Hz, peak_width_limits=(1, 100), 1–45 Hz, c) peak_width_limits=(1, 100), 1–95 Hz. Note that fooof fits the power line noise peak in a) and c) well.* *Supplementary to* [*Fig. 3 b)*](#kix.s23fpo4dpji6)

#
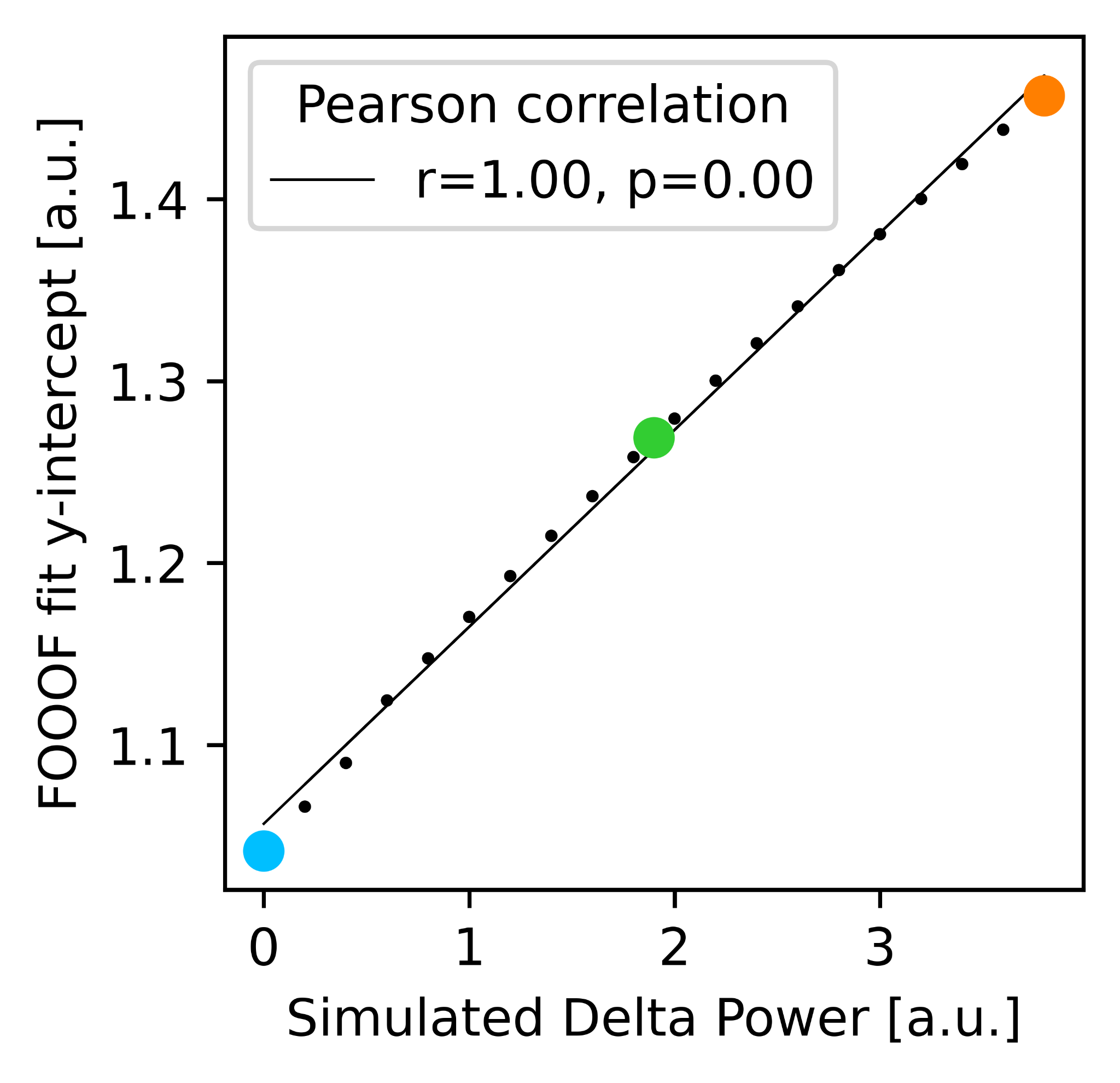


***SI*** ***Fig. 2*** *Periodic parameters impact aperiodic fits obtained with FOOOF. The y-intercepts of the FOOOF fits correlate with delta power. The blue, green, and orange data points correspond to the blue, green, and orange graphs of* [*Fig. 3 c)*](#kix.s23fpo4dpji6)*, the black data points indicate additional simulations.*


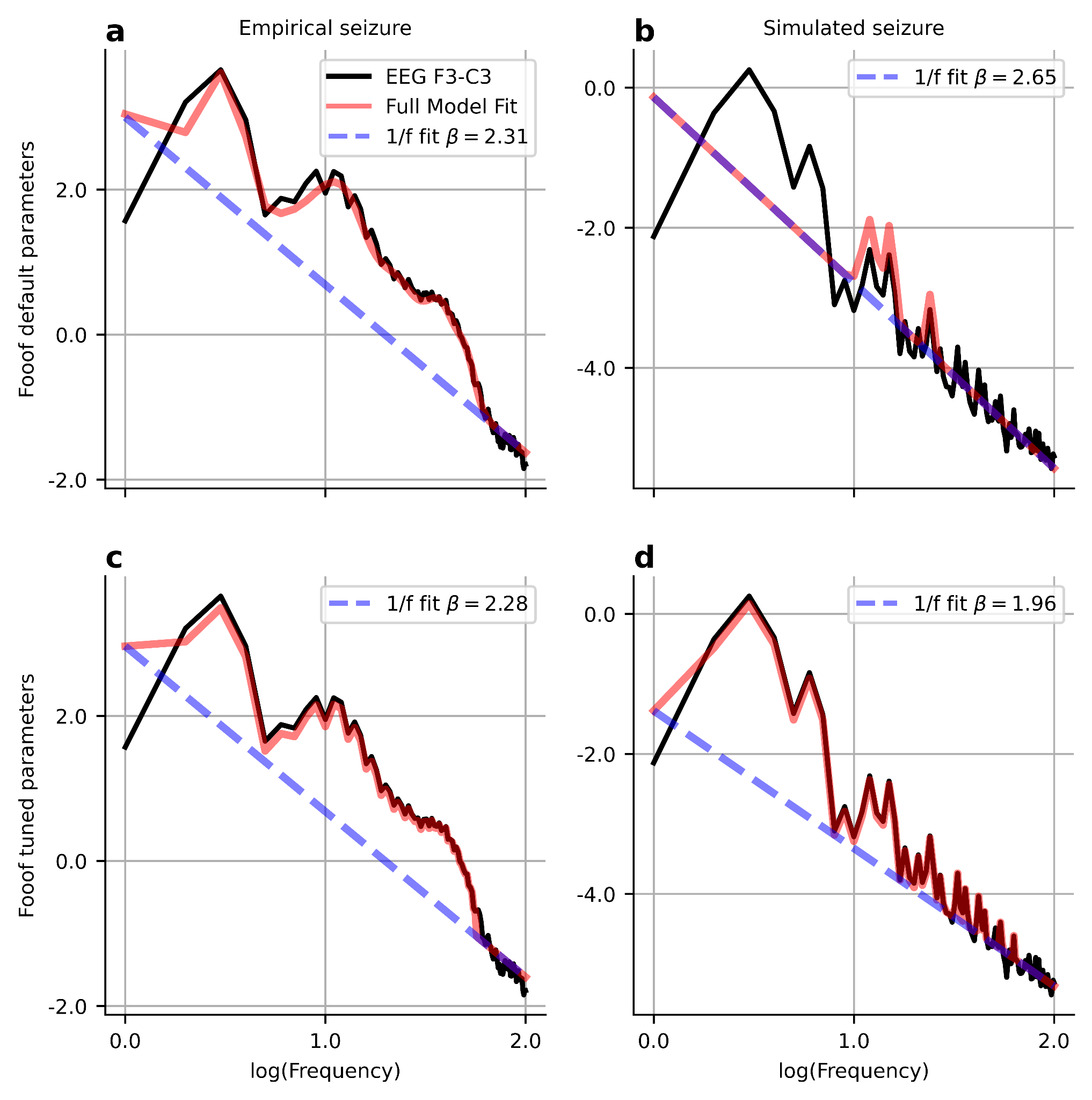


***SI*** ***Fig. 3*** *Overlapping peaks. a) FOOOF models the 3 Hz harmonics in the absence seizure recording as four peaks with center frequencies as 11 Hz, 22 Hz, 37 Hz, and 50 Hz. b) In the simulated seizure, three harmonic peaks are modeled as oscillations, whereas the rest is modeled as aperiodic component, leading to a large exponent of β=2.65 (ground truth β=1.8). c) and d) Even when the FOOOF parameters are tuned to allow maximum peak width limits of 1Hz (peak_width_limits=(0.5, 1)), FOOOF better models the harmonic peaks, but it still overestimates the 1/f exponent in the simulation. Supplementary to* [*Fig. 4*](#kix.1zx79kfavwin)


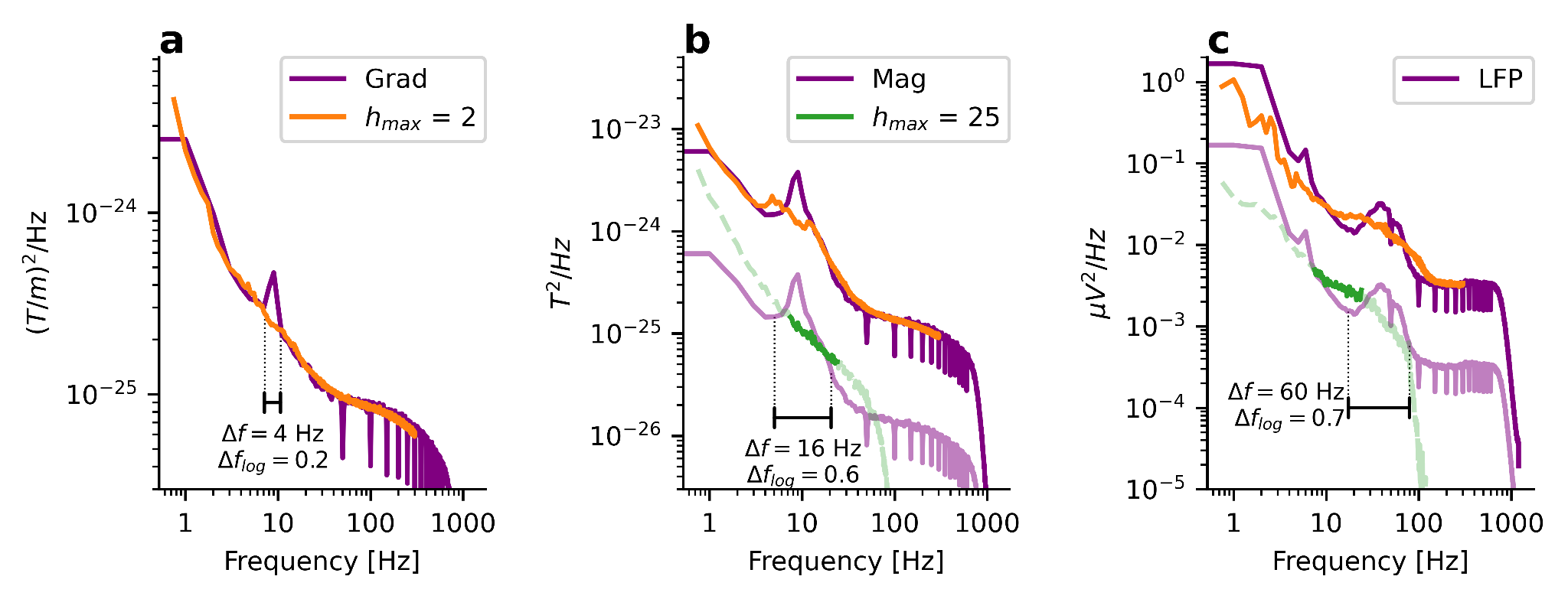


***SI*** ***Fig. 4*** *Large peak widths need large resampling factors. Dataset 3. a) MEG gradiometer PSD. A maximum resampling factor of 2 is sufficient to remove the alpha peak. b) MEG magnetometer PSD. For this peak width, a maximum resampling factor of 2 is not sufficient for removal. A maximum resampling factor of* $h_{max}=25$ *is sufficient but leads to a large evaluated frequency range. To avoid the high-pass and spectral plateau range, only a minor part of the aperiodic component (dark green) can be used for aperiodic fitting, whereas a major part is affected by the high-pass and spectral plateau (light green dashed). c) The same holds for the large beta peak in LFP data. Note that the logarithmic peak width is essential for setting the resampling factors, not the absolute peak width. All PSDs from dataset 3. Supplementary to* [*Fig. 6*](#kix.8we0jv7t1o37)


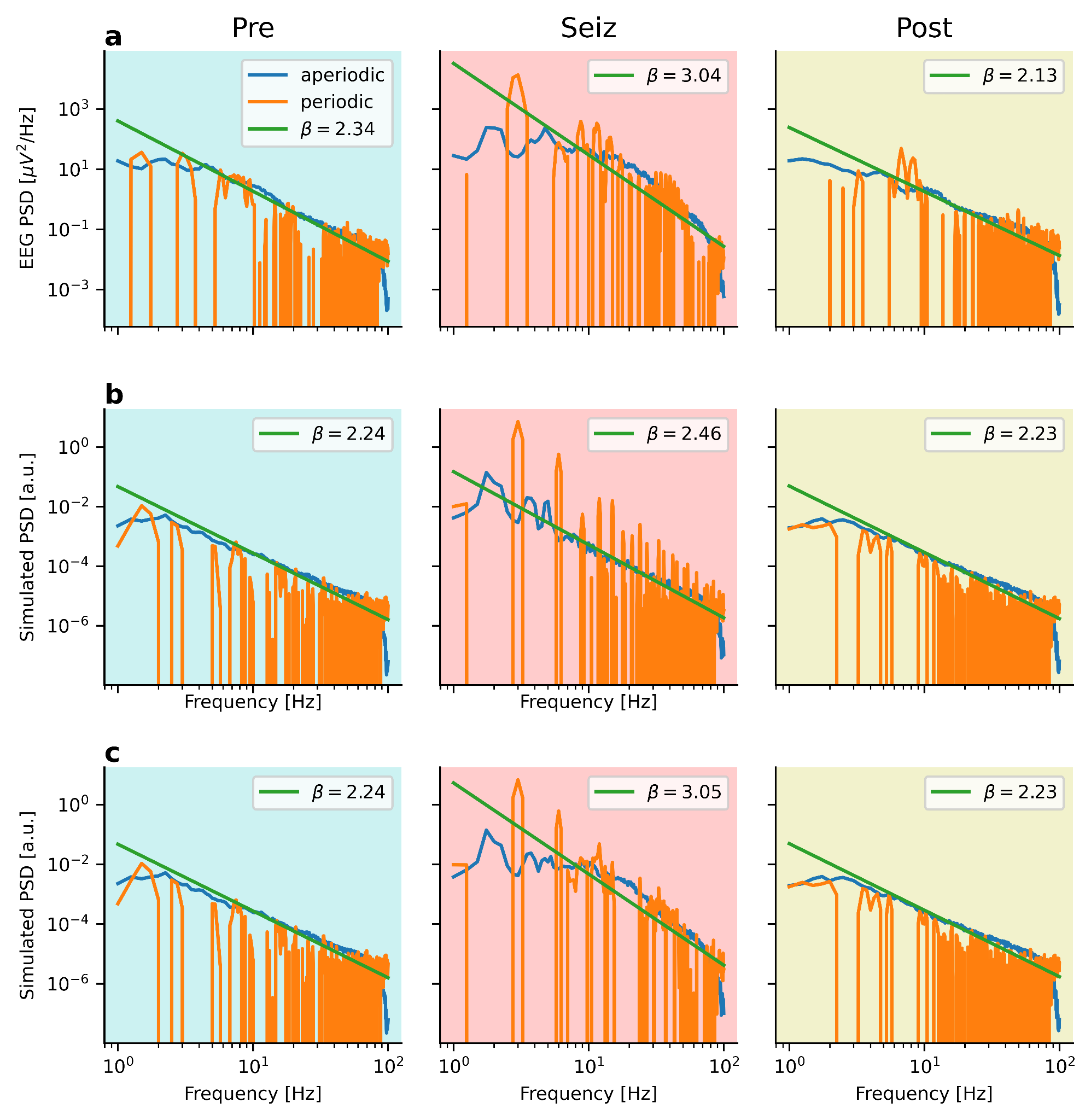


***SI*** ***Fig. 5*** *Overlapping peaks. Aperiodic (blue) and periodic (orange) extraction of IRASA and the corresponding 1/f fit (green) for the a) real and the b) and c) simulated time series in* [*Fig. 7*](#kix.1makq1pc89f2)*. For the simulation in b), IRASA can extract the harmonic 3Hz peaks well. However, the performance drops if two additional overlapping peaks are added. Supplementary to* [*Fig. 7*](#kix.1makq1pc89f2)
